# Supplementary material for: Integration of point-of-care screening for type 2 diabetes mellitus and hypertension with COVID-19 rapid antigen screening in Johannesburg, South Africa
Source: PLoS One. 2023 Jul 7;18(7):e0287794. doi: 10.1371/journal.pone.0287794 (PMC10328308; doi:10.1371/journal.pone.0287794)
Supplement: Attachment — Submitted filename: Reviewer responses (06012023).docx [file pone.0287794.s002.docx]

**Reviewer #1: The manuscript has improved and been clarified in the revised version. I just have some minor remarks regarding the results section about Predictors of diabetes, hypertension and linkage-to-care:**

We thank the reviewer for reviewing our manuscript a second time and providing useful feedback.

1. **As stated in the methods section and tables the predictors are about elevated blood glucose, elevated blood pressure and linkage-to-care and I suggest rephrasing the subheading accordingly.**

We thank the reviewer for their comment. We have made changes throughout the manuscript to make it clear that our study is assessing elevated blood glucose, blood pressure and linkage-to-care and not diabetes or hypertension.

2. **The last bit of the first sentence in the first paragraph seem to be missing.**

The sentence now reads, “The modified Poisson regression model we used to assess predictors of diabetes elevated blood glucose suggests that those >30 years of age, individuals with elevated blood pressure at enrollment (adjusted risk ratio (aRR) 1.96; 95% CI:1.15-3.33) and those with a BMI >25kg/m2 vs. <25kg/m2 (aRR 2.05; 95% CI:1.06-3.95) were at increased risk of elevated blood glucose (Table 2).

3. **As the 95% CIs are wide and include 1 indicating no effect, I suggest toning down the results described in the last sentence of the third paragraph in a similar way as the author have done with other non-significant findings. For example, an aRR of 0.68 with 95% CI 0.31–1.51 really means that individuals could be anywhere from 69% less likely to 51% more likely to seek care.**

We thank the reviewer for their comment. The sentence now reads, “We also found, although imprecise, that those with only elevated blood pressure at enrollment were less likely to seek care than those with only elevated blood sugar (aRR 0.68; 95% CI:0.31-1.51), while those with both elevated blood glucose and blood pressure were more likely to seek care compared to those with only elevated blood glucose (aRR 1.23; 95% CI:0.46-3.29).
